# Brain Network Signature of Autoscopic Phenomena in Humans

**DOI:** 10.1111/cns.70635

**Published:** 2025-10-29

**Authors:** Siyi Wang, Lei Qi, Xinqi Huang, Chunxue Wu, Jialin Du, Qing Xue, Jinghui Liu, Yuanhong Chen, Liankun Ren

**Affiliations:** ^1^ Department of Neurology, Xuanwu Hospital, Clinical Center for Epilepsy Capital Medical University Beijing China; ^2^ National Center for Neurological Disorders Beijing China; ^3^ Department of Neurology China Rehabilitation Research Center Beijing China; ^4^ Department of Radiology and Nuclear Medicine, Xuanwu Hospital Capital Medical University Beijing China; ^5^ Beijing Key Laboratory of Magnetic Resonance Imaging and Brain Informatics Beijing China; ^6^ Department of Pharmacy Phase I Clinical Trial Center, Xuanwu Hospital Capital Medical University Beijing China; ^7^ Jinan Hospital of Xuanwu Hospital Shandong First Medical University Jinan China

**Keywords:** autoscopic phenomena, epilepsy, functional human connectome, lesion network mapping, seizure semiology

## Abstract

**Aims:**

Autoscopic phenomena (AP) are distinct manifestations of epileptic seizure semiology and are also identified in diverse psychiatric disorders. Our study aimed to map the brain AP network and characterize its multimodal signatures.

**Methods:**

By performing lesion network mapping using the human connectome dataset (*n* = 1000), we investigated the brain AP network derived from 25 cases of AP caused by distributed focal brain lesions (*n* = 19) and direct electrical stimulation (DES) (*n* = 6). We further studied the multimodal signatures of the AP network, including exploring spatial correlation with human cortical developmental and evolutionary expansions, neurotransmitters, and electrophysiological rhythms.

**Results:**

We mapped a common AP network primarily involving the bilateral angular gyrus, posterior medial cortex, intraparietal sulcus, cuneus, fusiform and insula. Specifically, the bilateral thalamic pulvinar were identified as subcortical hubs. We further found a significant negative correlation between the AP network and developmental and evolutionary expansions. Particularly, the spatial density of norepinephrine transporter and the alpha frequency band power were significantly positively correlated with the AP network.

**Conclusion:**

Our study identified a common brain AP network and uncovered its multimodal signatures. These findings may clarify the network mechanisms underpinning AP, providing novel insights into bodily self‐consciousness.

## Graphical abstract

This study identified a common brain network underlying AP, encompassing bilateral angular gyrus, posterior medial cortex, intraparietal sulcus, cuneus, fusiform and insula, and uncovered its multimodal signatures. These findings advance our understanding of the network mechanisms of AP, as well as provide novel insights into bodily self‐consciousness.

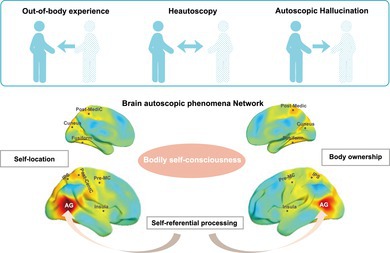

## 1 Introduction

Recognition of seizure semiology constitutes the cornerstone of epileptology since antiquity, as the characterization of its various forms continues to be a primary focus of contemporary research [1, 2, 3, 4, 5]. Of note, autoscopic phenomena (AP), classified among disorders of somatognosia, represent distinct manifestations of epileptic seizure semiology [6, 7]. To date, three classical forms of AP have been identified [8]: (1) out‐of‐body experiences (OBE), characterized by a sense of disembodiment, with the illusory body perceived at an elevated position relative to the physical body and attributed to self‐location; (2) heautoscopy (HAS), wherein self‐location is perceived simultaneously at both the physical and illusory bodies; and (3) autoscopic hallucination (AH), where the subject visualizes a second illusory body of their own, with self‐location typically anchored to the physical body. These phenomena share the common characteristic of visualized own body replications.

AP have been scarcely reported among epilepsy patients, yet evidences also indicate their presence in diverse psychiatric disorders including schizophrenia, depression, and anxiety [6, 7]. Indeed, AP embodies core scientific questions related to bodily self‐consciousness (BSC), which refers to the localization and attribution of the entire body or self to which the selected body part is attributed [9, 10]. Blanke et al. proposed that AP arise from aberrant integration of multisensory inputs pertaining to both personal and extrapersonal space, which represent the classical prototype of altered BSC [8]. Therefore, a better understanding of AP has profound implications for both fundamental and clinical significance.

AP have garnered considerable scholarly interest in recent decades. Scattered epileptic case reports of AP in association with brain lesions lend important insight into the neuroanatomical basis [6, 7]. Given that the involved brain regions were heterogeneously distributed among patients [7, 11], a complex but exquisitely integrated functional network rather than a single brain region contributing to AP is speculated. Although the brain networks of AP have been preliminarily explored, only focal lesions were used to calculate the AP network in prior studies [12]. Considering that AP can also be triggered by direct electrical stimulation (DES) [13, 14], the combination of lesion locations and DES sites will substantially strengthen the evidentiary basis for characterizing the brain AP network.

Most importantly, the multilayered organization of the brain AP network across micro, meso‐, and macro‐scale levels, remains unexplored yet. Previous studies have predominantly concentrated on simple components of the AP network, lacking in‐depth quantification and evaluation of neuroanatomical and functional features, which hampers a systematic understanding of the brain AP network. A promising strategy involves mapping micro‐scale properties, offering novel neurobiological perspectives. Emerging advanced methods integrate various neuroimaging techniques in time and space, and provide comprehensive insights into brain activity by integrating micro‐scale information and systems‐level dynamics [15, 16]. Molecular imaging technology allows us to observe changes in neurotransmitters, receptors, and proteins at a molecular level, enabling a more accurate interpretation of neuron activity details [17, 18, 19]. The multiscale estimate of interregional similarity gains insight into the brain's organizational structure, functional characteristics, and information processing mechanisms [18], enabling us to explore multilevel features within the AP network.

Here, using the technique of lesion network mapping (LNM) that initially identifies brain lesions functionally connected to a common neuroanatomical network [20, 21, 22] but has extended to atrophy [23, 24], activation [25, 26], and coordination‐based network mapping [27, 28], we mapped the large‐scale AP network at the whole brain level derived from lesions and stimulation sites using a wiring diagram of the human brain termed the human connectome (*n* = 1000) [29]. Moreover, utilizing the spatial mapping analysis, we investigated the multimodal signatures of the AP network. Characterizing the signature of the brain AP network may have significant clinical implications for AP, and provide new insights into BSC.

## 2 Methods

### 2.1 Literature Review and Case Selection

To identify AP caused by lesions or DES, we performed a systematic search on PubMed following PRISMA guidelines [30] with strict inclusion/exclusion criteria (Table S1). For each subgroup of AP, rigorous inclusion and exclusion procedures were implemented in compliance with the respective predefined definitions [8]. The common inclusion criteria for the three subgroups were as follows: (1) detailed case report: the availability of sufficient detail about the AP descriptions of the patients so that they could be classified with certainty, see more details in Table S2; (2) the AP form of the patient is definitely attributed to the focal structural or functional abnormality of the brain with accurate diagnosis, which is confirmed by imaging evidence such as magnetic resonance imaging (MRI) or CT or intracranial EEG records; (3) elicited by DES; (4) availability of a CT or MRI image depicting the lesions or stimulation sites of sufficient quality that could be facsimiled onto a standard brain template. The brain anatomical locations associated with certain symptoms were purified by exclusion shared by all three subgroups: (1) patients' descriptions of AP are often vague and ambiguous, making it challenging to delineate and accurately classify these experiences; (2) imaging data are either absent or of suboptimal quality in the literature, making it difficult to transform these data onto a standard brain template; (3) the patient with diffused lesions or the boundary of the lesions is hard to determine with the available image; (4) the patient with developmental AP or occurred in the postictal stage; and (5) the patient with any documented generalized disease, including meningitis, encephalitis, intoxications and psychiatric disorders.

This study was approved by the Ethics Committee of Xuanwu Hospital, Capital Medical University in accordance with the ethical standards of the Declaration of Helsinki, and informed consent was obtained from all patients.

### 2.2 Generation of the AP Network

Brain lesions were meticulously manually traced from published images or figures in 2D or 3D plane(s) reported in literature directly, utilizing neuroanatomical landmarks to accurately transfer the lesion regions onto a standard template brain from FSL (Montreal Neurological Institute (MNI) 152 2009b, 0.5 mm × 0.5 mm × 0.5 mm, http://fsl.fmrib.ox.ac.uk/fsldownloads/) using ITK‐SNAP 3.80 (http://www.itksnap.org/pmwiki/pmwiki.php) as has been widely acknowledged in previous work [31, 32, 33]. Although lesions were derived from images obtained across different studies, lesion delineation is fundamentally analyzed based on the neuroanatomical lesion localizations within the standard brain template. The results are not confounded by differences in imaging parameters across studies. The DES sites elicited AP were mapped to the template brain with the MNI coordinate. The coordinated nodes were created with a 3‐mm radius sphere centered on each stimulation site. The drawn masks (regions of interest, ROI) from all patients were used as individual seeds for further analysis.

The pipeline of LNM has been repeatedly validated in prior studies, including our recent work [21, 33, 34]. Briefly, to identify brain networks associated with AP, the large normative human connectome database of the Genomics Superstruct Project (GSP) from 1000 healthy subjects was used to compute the mean resting‐state functional connectivity (RSFC) of each patient's lesion in MNI152 space. The FC data are available online through the Harvard Dataverse at https://doi.org/10.7910/DVN/ILXIKS. The pipeline used to prepare the FC data is available at https://github.com/bchcohenlab/BIDS_to_CBIG_fMRI_Preproc2016. Pearson's correlation coefficient between the averaged time course of each lesion and the averaged time course of every voxel in the whole brain was calculated. A Fisher's *z* transformation was used to normalize these values resulting from 1000 subjects and transformed into a Fisher's *z* map. Then, a one‐sample *t*‐test was applied to determine the connectivity of lesions and DES sites causing AP. Positive correlations were thresholded at *T* > 7 [corresponding to family‐wise error (FWE)‐corrected *p* < 10^−6^] to create a binarized map of connected regions. The network overlay map for AP was thresholded to identify regions connected to > 80%. Using the same methodological approach, we also evaluated the spatial distribution patterns of brain networks across the three AP subgroups.

Next, to evaluate the specificity, the network map associated with AP was separately compared with the network maps of a control group (*n* = 68) that lesions causing other neurological symptoms derived from previous publications and our center's data (including 20 facial palsy, 17 amnesia, 19 mania, and 12 Parkinson's disease cases). Unthresholded lesion network maps for AP and control groups were compared using a voxel‐wise two‐sample *t*‐test in SPM 12, correcting for multiple comparisons with a conservative voxel‐based FWE rate *p* < 0.05.

To identify hubs for the AP network, the conjunction analysis was performed by multiplying the sensitivity analysis and the specificity testing. By definition, connectivity with the positive hub surviving from conjunction analysis defined a distributed brain network encompassing the lesions' locations causing AP and sites electing AP while avoiding control lesions.

To further verify the specificity of the AP network, ROI‐to‐ROI FC analysis was performed to quantitatively evaluate whether lesion locations or DES sites causing AP were more functionally connected to the positive hub surviving from conjunction analysis compared to the sites associated with other symptoms.

To assess the potential therapeutic relevance of the identified AP network, the deep brain hubs that were strongly connected to the network at the whole brain level were investigated.

### 2.3 Multimodal Signature of the AP Network

To characterize the multimodal features of the brain AP network, analyses were conducted using the neuromaps toolbox (https://github.com/netneurolab/neuromaps) [35]. We mapped the common brain AP network and these multimodal brain maps including cortical evolutionary and developmental expansions, neurotransmitter receptor and transporter maps, neurophysiological power maps to the Schaefer 100 parcellations in standard space. Subsequently, Fisher's *r*‐to‐*z* transform was applied to the functional connectivity coefficients within the AP network to normalize the data distribution for subsequent spatial correlation analysis [15, 36]. Pearson correlation analysis was then employed to assess the spatial correlation between the Fisher *z*‐transformed functional connectivity coefficients in the AP network (AP FZ values) and multimodal brain maps [33, 37, 38, 39].

### 2.4 Investigation of the Relation to Cortical Developmental and Evolutionary Expansions

We used the PALS‐TA24 developmental atlas proposed by Hill et al., which is a 24 population‐averaged, landmark‐and‐surface atlas derived from the hemispheres of full‐term infants and adults [40]. Within the PALS‐TA24 atlas, the cortical evolutionary expansion map, derived from the integration of functional and structural homology between the macaque monkey and the adult human, was registered. The surface area fractions of all positions in the two expansion maps were smoothed by 10 iterations (5 mm smoothing kernel) using an average neighbors algorithm [40]. A two‐sample *t*‐test was used to calculate the spatial correlation between the AP network and the developmental and evolutionary expansions of the cerebral cortex that were parcellated by Schaefer 100, respectively.

### 2.5 Delineation of the Neurotransmitter Receptors and Transporters Profiles

To quantify the relationship between neurotransmitters and the AP network, we acquired the positron emission tomography (PET) tracer images for neurotransmitter receptors and transporters from Hansen et al. and neuromaps (https://github.com/netneurolab/neuromaps) [35, 37]. Neurotransmitter maps included receptors/transporters that span 9 neurotransmitter systems, including dopamine (D1, D2, DAT), norepinephrine (NET), serotonin (5‐hydroxytryptamine receptor: 5‐HT1a, 5‐HT1b, 5‐HT2a, 5‐HT4, 5‐HT6, 5‐HTT), acetylcholine (α4β2, M1), glutamate (mGluR5), GABA (GABAa), histamine 3 (H3), cannabinoid receptor 1 (CB1) and mu‐opioid receptor (MOR). Each PET tracer image was registered to the MNI‐ICBM 152 nonlinear 2009 (version c, asymmetric) template, and segmented into 100 regions according to the Schaefer atlas. The spatial correlation was calculated between the AP network and these neuroreceptors' density maps.

### 2.6 Electrophysiological Fingerprint of the AP Network

The electrophysiological features were evaluated using MEG recordings across 6 canonical frequency bands from the Human Connectome Project (HCP, S900 release [41]), which were 6‐min resting‐state open‐eye time series from 33 healthy young adults in the age range 22–35 years. In brief, preprocessing was carried out by applying trap filters at 60, 120, 180, 240, and 300 Hz, followed by a high‐pass filter at 0.3 Hz to remove slow wave and DC offset artifacts, and co‐registered to individual structural MRIs using Brainstorm. Source reconstruction was conducted utilizing linearly constrained minimum variance (LCMV) beamforming, with time series parcellated according to the Schaefer‐400 atlas. MEG functional connectivity was assessed using amplitude envelope correlation (AEC), which was determined as the absolute value of the Hilbert transform of band‐limited cortical source activity for each defined frequency band [42, 43, 44]. AEC was calculated between all pairs of brain regions across six frequency bands (delta: 2–4 Hz, theta: 5–7 Hz, alpha: 8–12 Hz, beta: 15–29 Hz, low gamma: 30–59 Hz, high gamma: 60–90 Hz), which was parcellated to 100 cortical regions. The composite connectivity metric was derived by extracting the first principal component from the set of frequency‐specific connectivity matrices, and normalization for comparison was achieved by applying Fisher's *r*‐to‐*z* transformation [42, 45]. Spatial Pearson's correlations were assessed between the AP network and each frequency band.

### 2.7 Statistical Analysis

Prior to analysis, the normality of all data was evaluated using the Shapiro–Wilk test. Data exhibiting a normal distribution (*p* > 0.05 on the Shapiro–Wilk test) were analyzed using parametric tests. For data that did not meet the criteria for a normal distribution, the appropriate nonparametric tests were used. The corresponding statistical analyses were described in the data processing procedures in Section 2. A one‐sample *t*‐test was then applied to determine the connectivity of lesions leading to AP. A two‐sample, two‐tailed independent means *t*‐test was used to compare the unthresholded derived functional network maps associated with AP and the control group, with conservative voxel‐wise FWE correction for multiple comparisons at *p* < 0.05. Pearson correlation was used to calculate the spatial correlation between the AP functional network and the multiscale brain maps.

## 3 Results

### 3.1 Included Subject Characteristics

All data used in the present work, including neuroimaging data of focal brain lesions and the stimulation sites, were collected from existing published literature.

**FIGURE 1:**
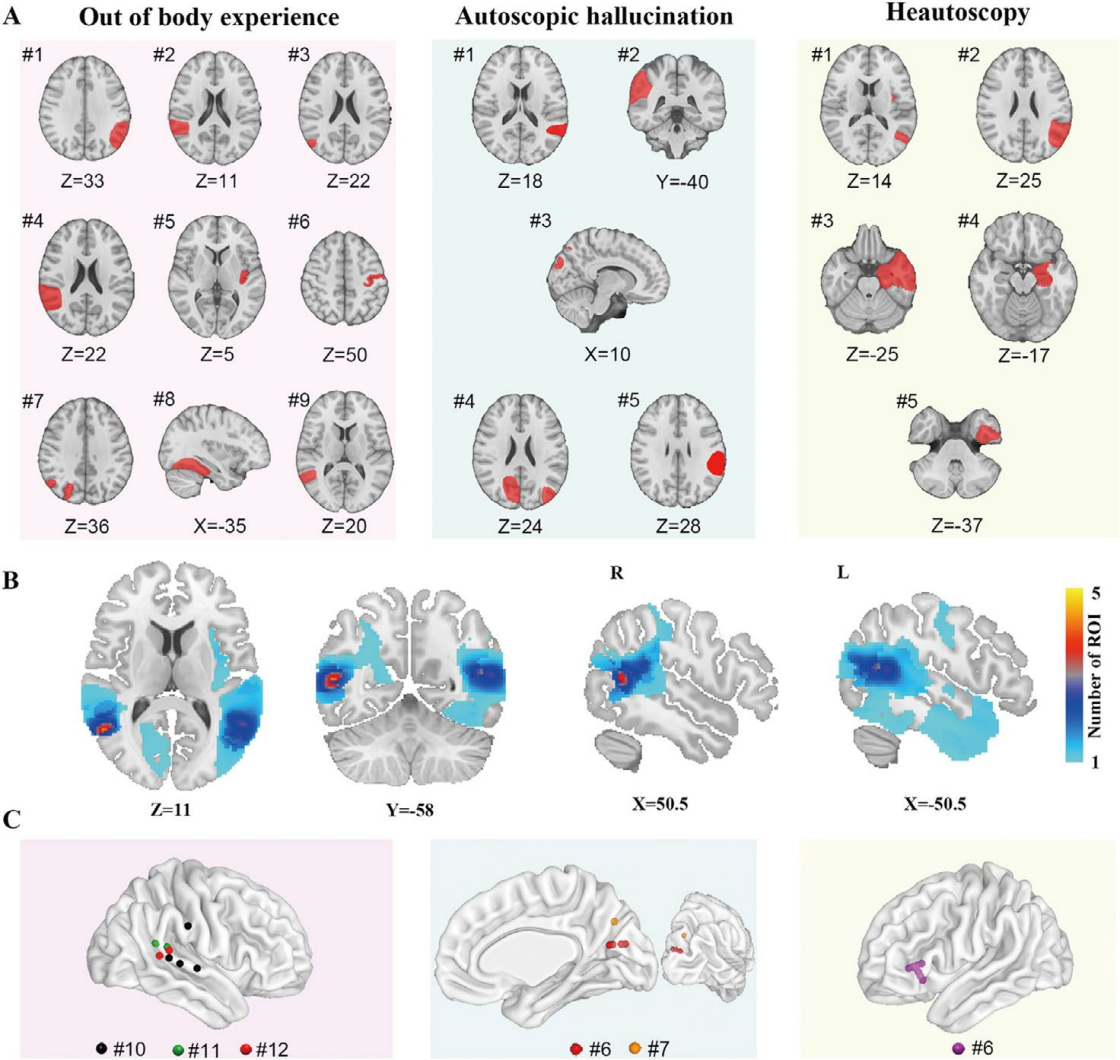
Corresponding brain lesions or stimulation sites associated with AP distributed in distinct brain regions. For each AP subtype, the representative slices of lesions (red) and spherical ROI of the stimulation sites (radius = 3 mm) were displayed on a standard human brain template for each patient. (A) Brain lesion sites associated with different subtypes of AP were manually recorded into standard brain templates (OBE: *n* = 9, AH: *n* = 5, HAS: *n* = 5). (B) All brain lesions associated with AP were registered onto a common standard brain template to facilitate further analysis and then assigned ROI numbers. (C) DES sites elicited different AP were displayed in the standard brain template. AH, autoscopic hallucination; AP, autoscopic phenomena; DES, direct electrical stimulation; HAS, heautoscopy; OBE, out‐of‐body experience; ROI, region of interest.

We identified a total of 25 patients who reported AP by a literature search of PubMed following PRISMA flow with strict inclusion and exclusion criteria (Table S1). Specifically, 12 patients showed out‐of‐body experience (nine patients had definite structural lesions and three patients were induced by DES); seven patients showed self‐hallucination (five patients had definite structural lesions and two patients were induced by electrical stimulation); six patients showed self‐observation in vitro (five patients had definite structural lesions, and one patient was induced by electrical stimulation), as shown in Figure 1.

### 3.2 Definition of the Common Brain AP Network

**FIGURE 2:**
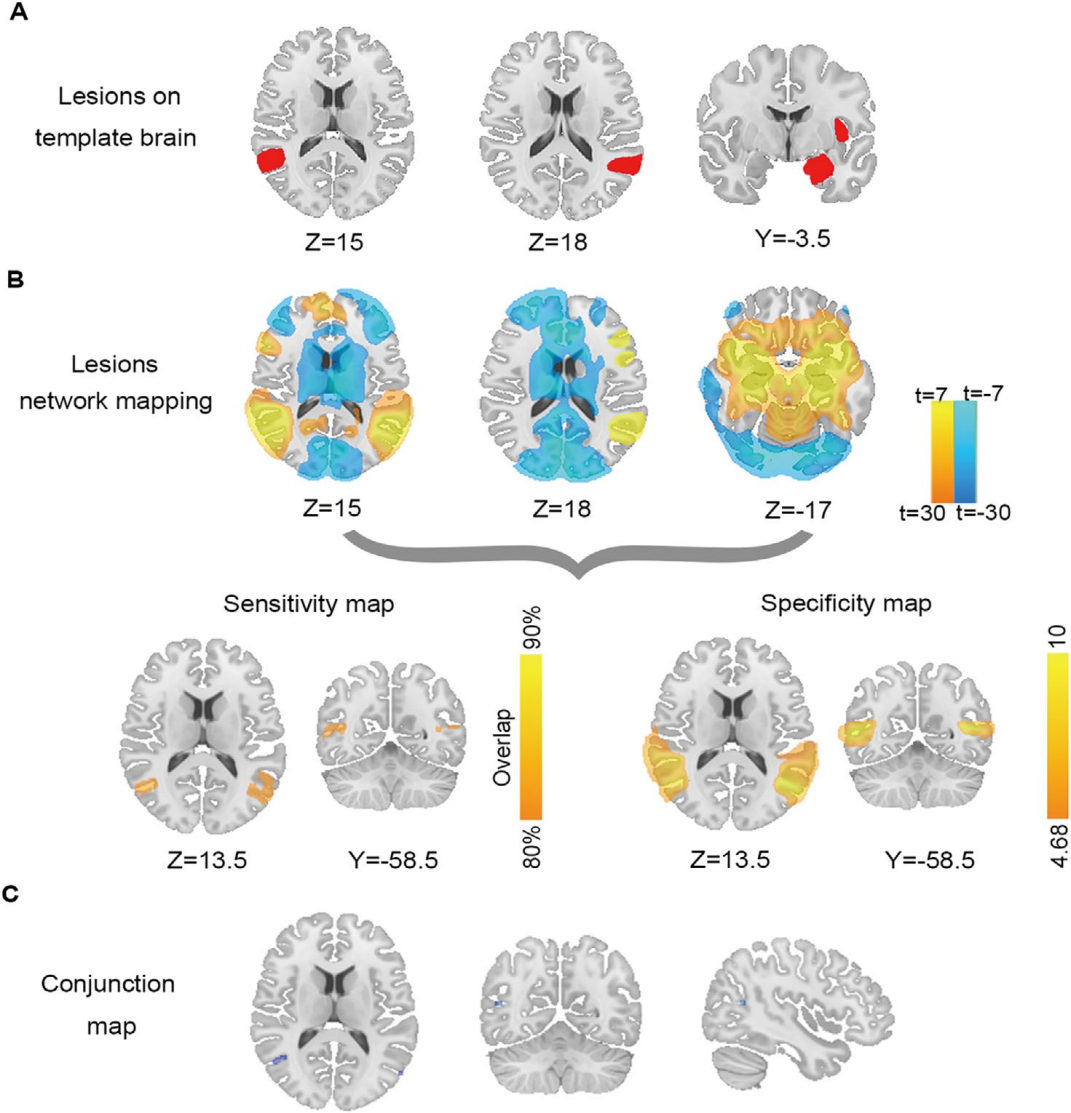
Protocol of lesion network mapping. (A) Representative lesions causing AP (including OBE, HAS, and AH) were mapped on the template brain. (B) Using the Brain Genomics Superstruct Project (*n* = 1000), the brain regions functionally connected to each brain lesion were identified. The network‐associated AP was derived from both lesions and stimulation site coordinates. Overlap of specificity tests used to identify connections specific to lesions and stimulation site locations causing AP versus the control group (other symptoms). (C) The network hubs with both sensitivity and specificity associated with AP were identified by conjunction analysis. AH, autoscopic hallucination; AP, autoscopic phenomena; HAS, heautoscopy; OBE, out‐of‐body experience.

We evaluated overlap in lesion‐based and DES sites‐based resting state networks. As a result, 80% (20/25) of these locations were positively connected to the bilateral angular gyrus (AG) with a T‐threshold of seven (corresponding to voxel‐wise FWE‐corrected *p* < 10^−6^), as shown in Figure 2A,B. Also, bilateral AG was detected by the specificity test in comparison with the network maps of a control group (*n* = 68) that had lesions causing other neurological symptoms (FWE‐corrected *p* < 0.05) (Figure 2B). The specific regions over the bilateral AG were identified by the conjunction analysis of the AP network map (Figure 2C).

**FIGURE 3:**
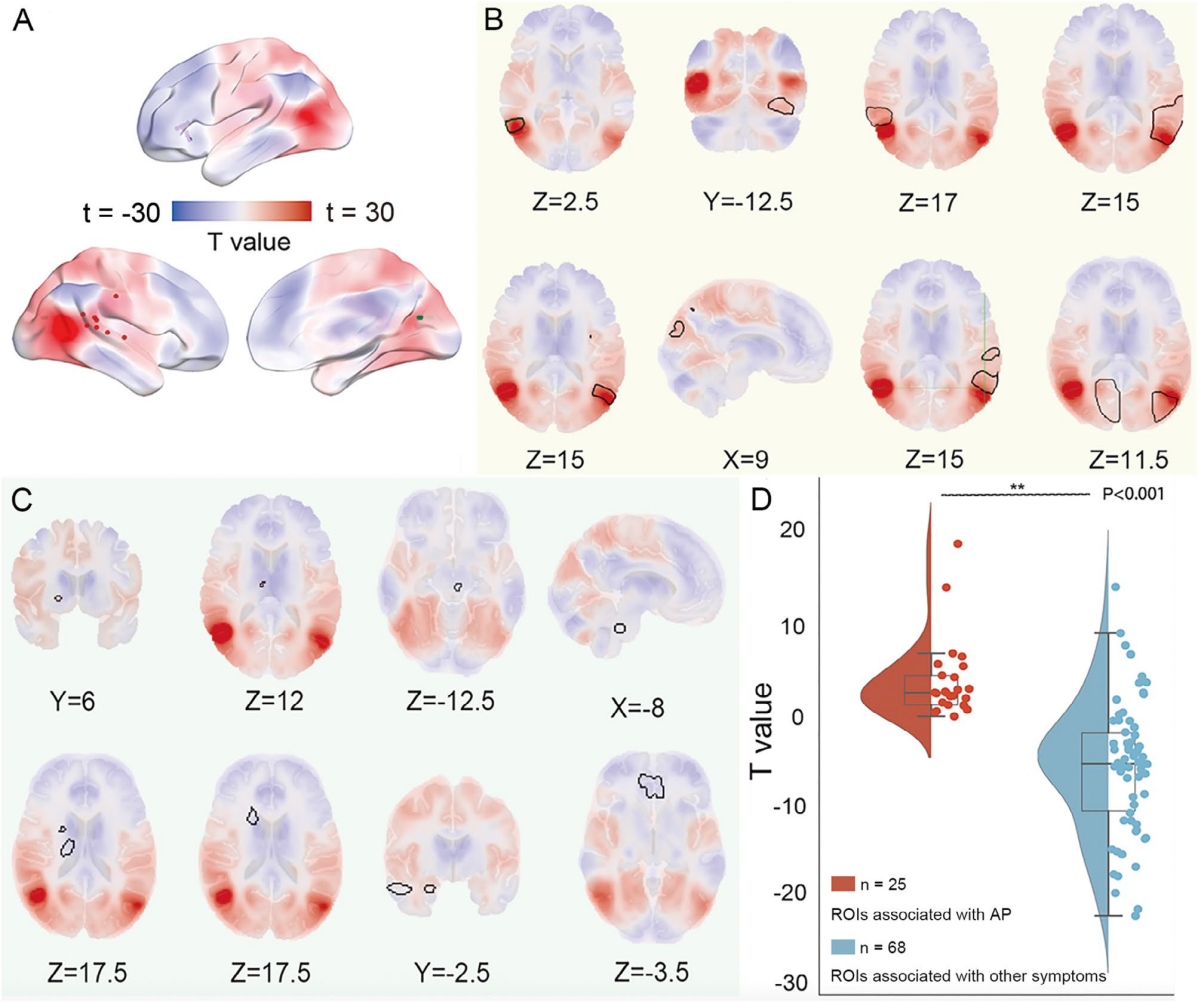
The common brain AP network and its specificity. (A) The AP network comprises brain regions that are specifically associated with AP‐related ROIs rather than other symptoms. (B, C) The defined AP network encompass the heterogenous locations causing corresponding symptoms but could not encompass the locations causing other symptoms of the control group, (B) ROIs associated with AP, and (C) ROIs associated with other symptoms. (D) The *t*‐test compares the connectivity of ROIs associated with AP versus those associated with other symptoms (voxel‐wise FWE‐corrected *p* < 0.05). ROI, region of interest.

Then, the identified network hubs were binarized to derive the underlying brain network with positive FC of certain AP using the same pipeline as the method. The resulting network t maps were transformed into Fisher z maps. We shall refer to this common network as the human brain AP network. Finally, the brain AP network consists of bilateral AG, posterior medial cortex (post‐MediC), intraparietal sulcus (IPS), premotor cortex (pre‐MC), somatosensory cortex, cuneus, fusiform and insula (Figure 3A–C). As expected, lesions and DES sites associated with AP aligned well with the common AP network, while lesions associated with other symptoms did not (Figure 3A–C). The results of ROI‐to‐ROI FC analysis indicated that the sites associated with AP were more connected (two‐sample *t*‐test, *p* < 0.05, Figure 3D) to the centered hub in bilateral AG compared to the sites unrelated to AP.

In addition, our mapping of the brain network of the respective subgroup showed a high degree of similarity in spatial distribution among OBE, AH, and HAS, all of which closely mirrored the distribution of the overall AP network (Figure S1).

### 3.3 Therapeutic Relevance for Deep Brain Stimulation

**FIGURE 4:**
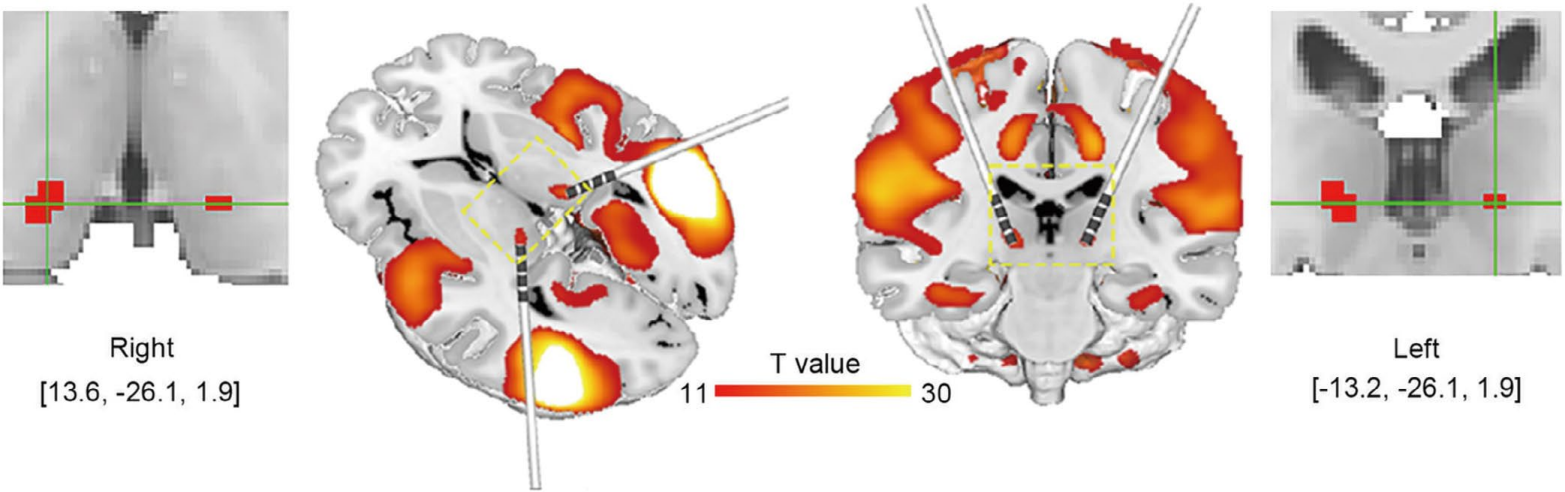
Subcortical hubs of the AP network. Bilateral hubs over pulvanar were identified as semiology‐specific targets for deep brain stimulation.

The AP network involved peak FC to bilateral thalamic pulvinar, which were identified as subcortical hubs of the AP network, as evidenced by *T*‐values > 11 (Figure 4). The results suggest that the bilateral thalamic pulvinar may serve as the central hub in the integration and transmission of information within the AP network, thereby representing a promising target for network‐based neuromodulation.

### 3.4 Association Between the AP Network and Cortical Developmental and Evolutionary Expansions

We investigated the relevance of the AP network to human developmental and evolutionary expansions to test whether the organization of the AP network is along the developmental and evolutionary axes.

**FIGURE 5:**
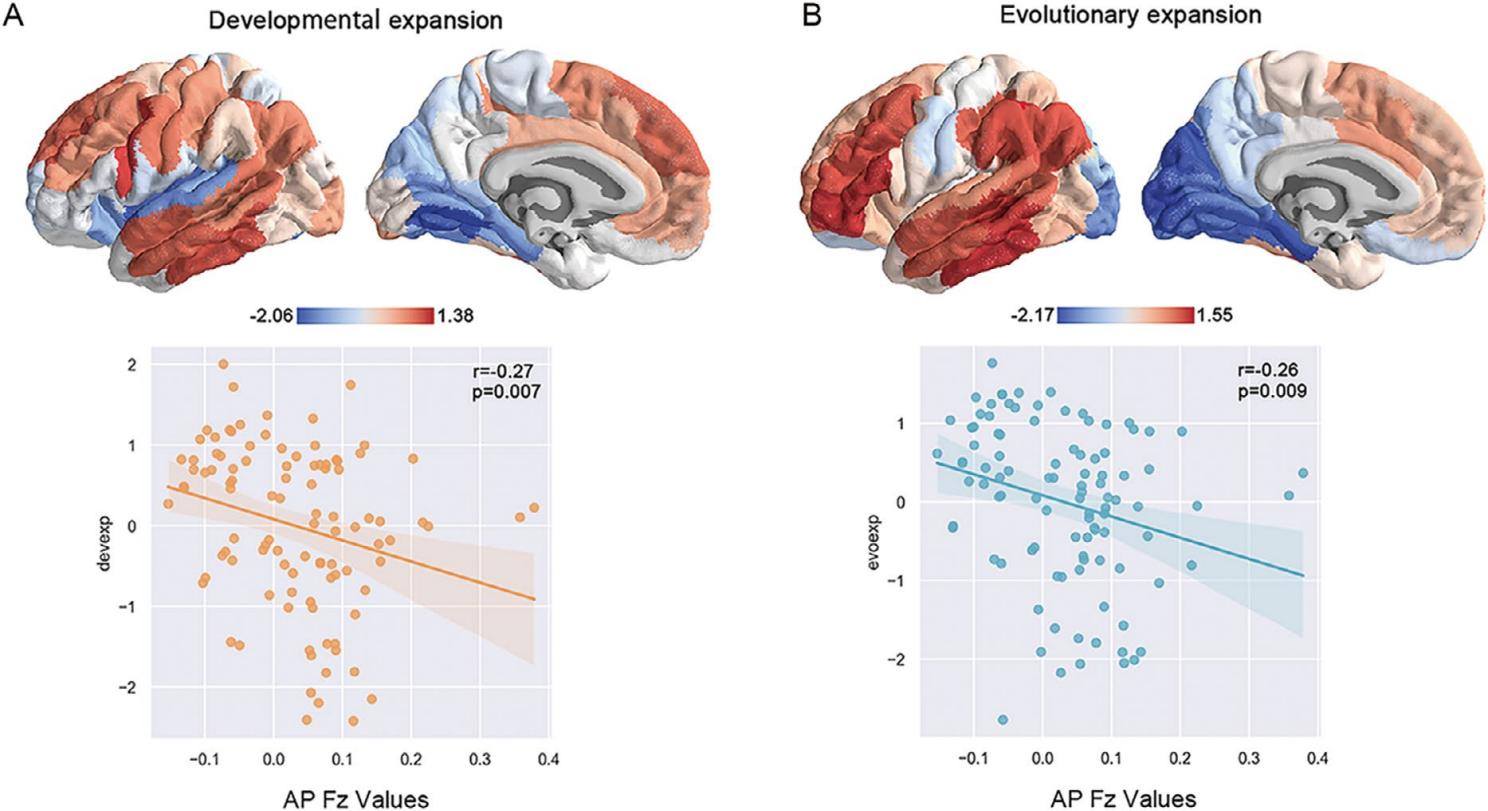
The correlation between the AP network and cortical developmental, evolutionary axes. The AP network showed a negative correlation with both (A) developmental and (B) evolutionary expansions, the relevant trends are very similar.

The patterns of the cortical developmental and evolutionary expansions exhibited striking similarities. Low‐expansion regions are primarily concentrated in the medial temporal, cuneus, and occipital regions in two maps, which are typically associated with early‐onset maturation [40]. Aligning with established neurodevelopmental and evolutionary trajectories, our data unveiled that AP Fz values were negatively correlated with both developmental expansion (*r* = −0.27, *p* = 0.007) and evolutionary expansion (*r* = −0.26, *p* = 0.009) (Figure 5A,B).

### 3.5 Norepinephrine Transporter Density Positively Influences the AP Network

**FIGURE 6:**
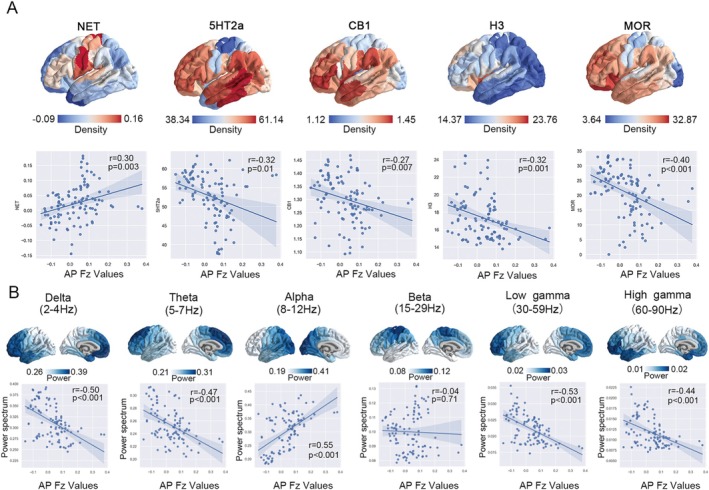
The correlation between the AP network and different neurotransmitter receptors and transporters, and neural oscillations. (A) Different types of neurotransmitter receptors and transporters density and the AP network presented different correlations, with NET manifesting a specific positive correlation (*r* = 0.30, *p* = 0.003, FDR). (B) Different frequency bands exhibited different correlations with the AP network, and a significant positive correlation (*r* = 0.55, *p* < 0.001, FDR) was observed in the alpha band (8–12 Hz). 5‐HT, serotonin receptors: 5‐hydroxytryptamine receptor; CB1, cannabinoid receptor 1; D1, D2, DAT, dopamine receptors; H3, histamine 3; MOR, mu opioid receptor; NET, norepinephrine transporter; α4β2, acetylcholine receptor: A2B2; mGluR5, glutamate receptor.

We further evaluated the effect of spatial variation in the density of different neurotransmitters on the AP network. The result showed that five of these neurotransmitter receptors and transporters (NET, 5‐HT2a, CB1, H3, and MOR) showed a significant correlation to the AP network. Notably, only the density of NET (*r* = 0.30, *p* = 0.003) exhibited a positive correlation with the AP network, while the remaining receptors demonstrated negative correlations, including 5‐HT2a (*r* = −0.32, *p* = 0.01), CB1 (*r* = −0.27, *p* = 0.007), H3 (*r* = −0.32, *p* = 0.001), and MOR (*r* = −0.40, *p* < 0.001) (Figure 6A).

### 3.6 AP Network Specific Neural Oscillations

We assessed the relationships between the AP network and each frequency electrophysiological network. A pronounced elevation of alpha power is localized to the human occipital cortex. Notably, our analysis revealed a positive correlation between the spatial distribution of the alpha band (*r* = 0.55, *p* < 0.001) and the AP network (Figure 6B). The results indicated a region‐specific modulation of neural oscillatory activity, suggesting a potential unique electrophysiological signature of the AP network.

## 4 Discussion

We mapped a common brain network of AP and systematically investigated the corresponding brain network signatures. Several critical insights have been unveiled. Primarily, we have delineated a common network underpinning AP that is characterized by pivotal nodes situated within the bilateral AG. Of note, bilateral thalamic pulvinar sites were identified serving as potential therapeutic targets for brain stimulation. Our data further revealed a spatially negative correlation between the AP network and the processes of developmental and evolutionary expansions. Intriguingly, the spatial distribution of NET density and alpha band oscillation power has a positive correlation with this AP functional network.

AP subgroups demonstrated a high degree of similarity in spatial distribution and were remarkably consistent with the overall AP network, pointing to a common brain AP network. The brain network of AP we identified is predominantly connected bilaterally to the superior parietal gyrus extending to IPS, post‐MediC, the somatosensory cortex, the pre‐MC, cuneus, fusiform, and insula, primarily centered around the bilateral AG with a right hemispheric predominance. Indeed, the functions of all the structural components have been previously well characterized. AG is well known to play a crucial role in visual–spatial localization, perspective transformation, and multimodal information integration [46]. Administration of dissociative agents was found to activate post‐MediC in mice [47]. Investigations using fMRI and PET have suggested that activation of bilateral IPS, somatosensory cortex, and insula is associated with illusory bodily ownership [9, 48]. Pre‐MC primarily controls motor planning and execution. Furthermore, the cuneus and fusiform gyrus are not only limited to visual processing but are also involved in multisensory integration and cognitive processing [49]. Together, AP are involved in multisensory processing functions of integrating somatosensory, visual, vestibular, and interoceptive signals [50].

Considering the formation of BSC remains a matter of scientific debate, our findings also offer neurobiological insights into BSC. Our results found that the AP network is negatively correlated with developmental and evolutionary expansions, suggesting that BSC might be a primitive function in the evolutionary process, rather than a sophisticated attribute unique to humans, consistent with established findings [51, 52]. In the temporal sequence of human cortical development, the sensorimotor regions within the AP network demonstrate early maturation, which may be unsusceptible to external interventions [53].

Specifically, symptom‐specific network mapping may help identify specific regions that could be targeted for alleviating the symptom. Over recent years, deep brain stimulation (DBS) has been recognized as a promising approach to treat a range of brain disorders [54, 55]. Nevertheless, the outcomes remain heterogeneous [56, 57]. Refining optimal targets for brain stimulation remains critical for therapeutic efficacy. Previous resting‐state network studies have demonstrated a significant correlation between the optimal sites for DBS and those identified for noninvasive stimulation, suggesting that both invasive and noninvasive interventions may modulate the same brain network, providing a theoretical framework to guide the identification of DBS therapeutic targets through the FC network [58]. According to the anatomico‐electro‐clinical principles, the selection of DBS targets is fundamentally anchored in seizure semiology [59]. By identifying the peak FC target within the AP network, our study revealed that the bilateral thalamic pulvinar functions as pivotal subcortical hubs of the AP network, offering potential therapeutic significance.

Our data characterized the specific micro‐architecture of the AP network. The positive correlation between NET and the AP network suggests that NET may demonstrate a regionally selective enrichment and contribute to the integration of the AP network. At the cortical level, NET exerts regulatory effects on cognitive processes, emotional responses, and attentional functions. Dysregulation of NET has been linked to neurological disorders such as depression and attention deficit hyperactivity disorder [60]. Conversely, the neurotransmitters 5‐HT2a, CB1, H3, and MOR demonstrated significant negative correlations with the AP network, indicating their nonspecific role with respect to this network. Meanwhile, by correlating regional neurophysiological dynamics across the human brain, a specific network synchronized rhythm (8–12 Hz) of the AP network was revealed, suggesting alpha oscillations may represent the intrinsic electrophysiological characteristics of the AP network. These findings revealed the multimodal signatures of the AP network, paving the avenue to the development of precision pharmacotherapies targeting specific neurotransmitters, as well as neuromodulation based on region‐specific neural oscillatory dynamics within brain networks.

Undeniably, several potential limitations should be noted. Firstly, the present case reports were identified through a systematic literature review. The possibility of publication bias remains unavoidable. Secondly, a large normative human brain connectome was used to assess functional connectivity. Given that lesions might alter patients' brain functional connectome, further studies utilizing personalized data are needed. In addition, considering that the multimodal brain maps employed in our spatial correlation analyses are derived from population‐level datasets, individual differences may not be fully captured. Future investigations incorporating individual‐level validation will be essential to enhance the robustness of these findings.

## 5 Conclusions

Overall, our findings may advance the understanding of the network mechanisms of AP and hold potential clinical therapeutic implications for AP, offering novel perspectives on bodily self‐consciousness.

## Author Contributions

**Siyi Wang:** investigation, data curation, formal analysis, writing – original draft preparation, visualization. **Lei Qi:** investigation, data curation, formal analysis, writing – original draft preparation, visualization. **Xinqi Huang:** investigation, data curation, formal analysis, writing – original draft preparation, visualization. **Chunxue Wu:** investigation, data curation, formal analysis, validation. **Jialin Du:** investigation, data curation, formal analysis, validation. **Qing Xue:** investigation, data curation, validation. **Jinghui Liu:** investigation, data curation, validation. **Yuanhong Chen:** investigation, data curation, validation. **Liankun Ren:** conceptualization, supervision, writing – review and editing, funding acquisition, project administration, resources. The authors read and approved the final manuscript.

## Ethics Statement

This study was approved by the Ethics Committee of Xuanwu Hospital, Capital Medical University.

## Conflicts of Interest

The authors declare no conflicts of interest.

## Supporting information


**Table S1:** PRISMA flow diagram.
**Table S2:** The detailed information of AP patients.
**Figure S1:** Lesion network mapping of OBE, AH, and HAS.

## Data Availability

All datasets included neuroimaging data of focal brain lesions and the stimulation sites used in the present work were collected from existing published literature, which has been included in the Supporting Information. The code used for data processing, electrode reconstruction, and data visualization is openly available from public toolboxes (https://www.leaddbs.org; https://fsl.fmrib.ox.ac.uk/fsl/fslwiki; https://www.fil.ion.ucl.ac.uk/spm/software/spm12; https://stnava.github.io/ANTs; https://github.com/netneurolab/neuromaps).
