# Supplementary material for: Brain Network Signature of Autoscopic Phenomena in Humans
Source: CNS Neurosci Ther. 2025 Oct 29;31(11):e70635. doi: 10.1111/cns.70635 (PMC12571770; doi:10.1111/cns.70635)
Supplement: Supplementary file 1 — Table S1: PRISMA flow diagram. Table S2: The detailed information of AP patients. Figure S1: Lesion network mapping of OBE, AH, and HAS. [file CNS-31-e70635-s001.docx]

**Supplementary Materials**

**Table S1** **PRISMA flow diagram**


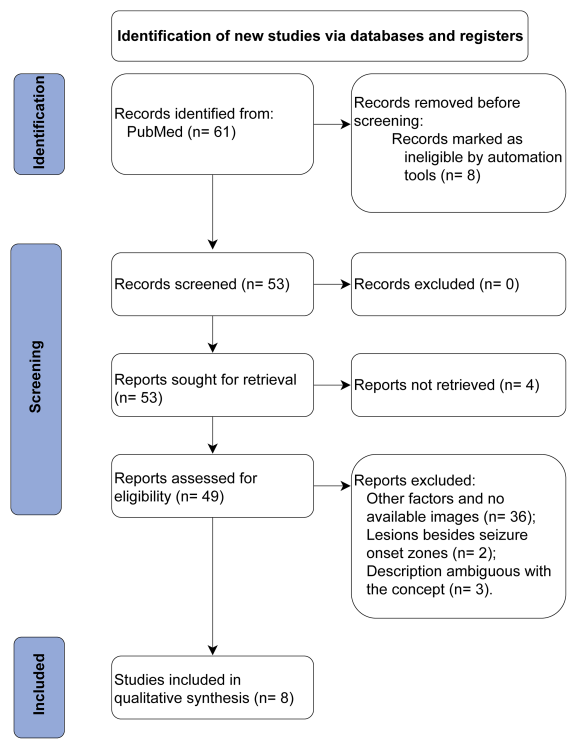


**Table S2 The detailed information of AP patients**

| Patient number | Age | Gender | Dominant hand | Evoked symptom | Lesion/Stimulation | Literature | Lesion site /Mapping site | Original image |
| --- | --- | --- | --- | --- | --- | --- | --- | --- |
| 1 | 22 | female | right-handed | OBE#1 | lesion | Blanke O, et al, Brain, 2004,#2 & cited by I Ionta S, et al, Neuron, 2011 #5 | L_parieto-temporal cortex | 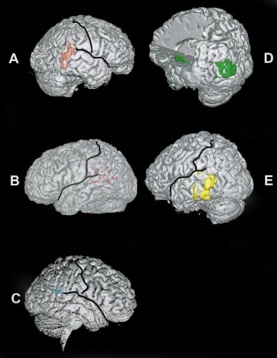 |
| 2 | 46 | female | right-handed | OBE#2 | lesion | Blanke O, et al, Brain, 2004,#1 & cited by I Ionta S, et al, Neuron, 2011 #8 | R_occipito-parietal dysembryoblastic neuroepithelial tumor | 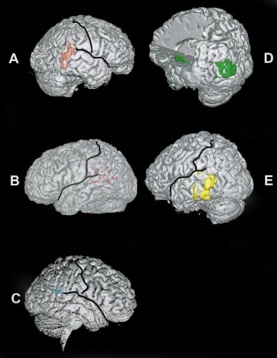 |
| 3 | 44 | male | no available | OBE#3 | lesion | Brandt C, et al, Nervenarzt. 2005& cited by I Ionta S, et al, Neuron, 2011 #3 | R_angular gyrus | 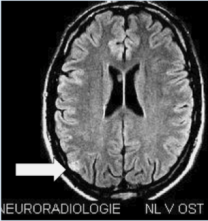 |
| 4 | 10 | boy | right-handed | OBE#4 | lesion | Heydrich L,et al, Epilepsy Behav. 2011 | R_angular gyrus, inferior parietal lobe, anterior mesiotemporal lobe | \| \| 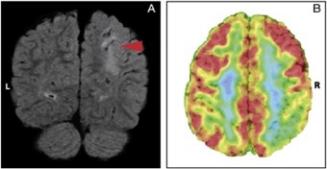 \| \| --- \| \| \| --- \| --- \| |
| 5 | no available | no available | no available | OBE#5 | lesion | Hoepner R, et al, J Neurol. 2013, #1 | L_posterior insula | 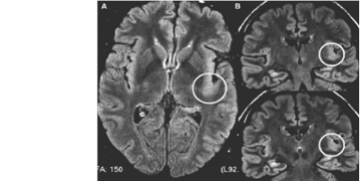 |
| 6 | no available | no available | no available | OBE#6 | lesion | Hoepner R, et al, J Neurol. 2013, #2 | L_pre- and post-central gyrus reaching the TPJ | 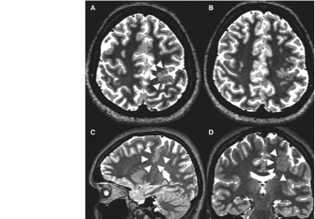 |
| 7 | no available | no available | no available | OBE#7 | lesion | Hoepner R, et al, J Neurol. 2013, #3 | R_parietal & extending to angular gyrus | 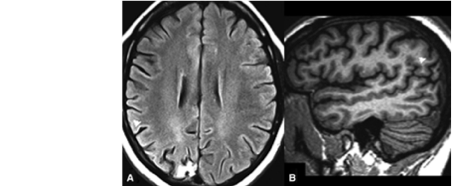 |
| 8 | no available | no available | no available | OBE#8 | lesion | Hoepner R, et al, J Neurol. 2013, #5 | L_fusiformegyrus | 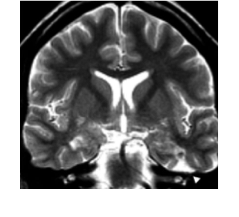 |
| 9 | 15 | boy | right-handed | 0BE#9 | lesion | Tie Fang et al, J Neurosurg Pediatric, 2014 | no available | 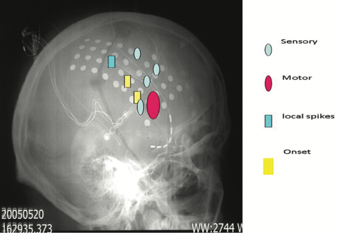 |
| 10 | no available | no available | no available | OBE #10 | stimulation | Penfield, W. and Rasmussen, T. (1950) The Cerebral Cortex of Man,Chapter IX；cited by Tong F. Trends Cogn Sci. 2003 | no available | 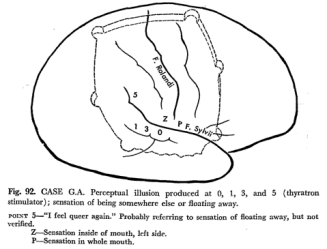 |
| 11 | 45 | female | right-handed | OBE #11 | stimulation | Blanke O,et al, Nature. 2002 | R_angular gyrus | 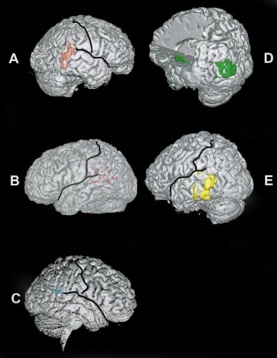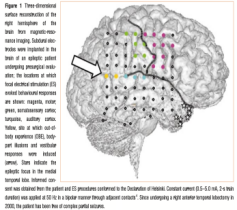 |
| 12 | 63 | male | no available | OBE #12 | stimulation | De Ridder, et al, N Engl J Med.2007 & cited by I Ionta S, et al, Neuron, 2011 #6 | no available | 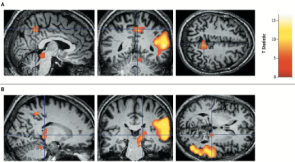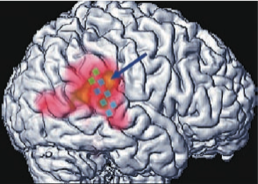 |
| 13 | 37 | female | right-handed | AH#1 | lesion | Blanke O, et al, Brain, 2004,#6 | L_posterior parts of the superior & middle temporal gyrus, and the adjacent angular gyrus | 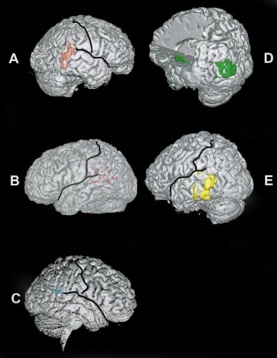 |
| 14 | 36 | female | right-handed | AH#2 | lesion | Maillard et al., 2004, #1;cited by Heydrich L, et al, Brain. 2013,#2 | R_inferior parietal gyrus | 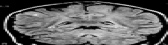 |
| 15 | 27 | female | right-handed | AH#3 | lesion | ﻿﻿ Blanke O, et al, Epileptologie. 2007;cited by Heydrich L, et al, Brain. 2013,#4 | bilateral occipital (e.g. right mesial occipito-parietal cortex) | 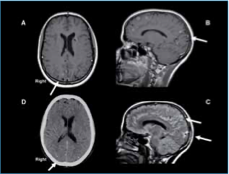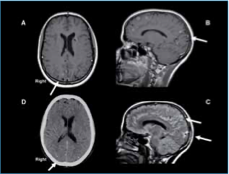 |
| 16 | no available | no available | no available | AH#4 | lesion | Hoepner R, et al, J Neurol. 2013, #4 | no available | 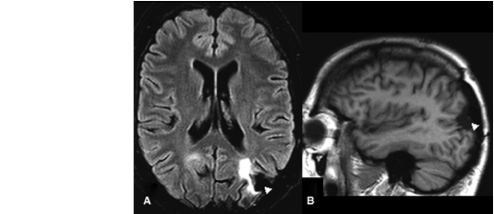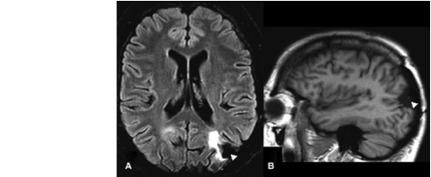 |
| 17 | 14 | female | right-handed | AH#5 | lesion | ﻿Fonti D etal, Epileptic Disorders,2021 | bilateral polymicrogyric cortex in the supramarginal gyrus | 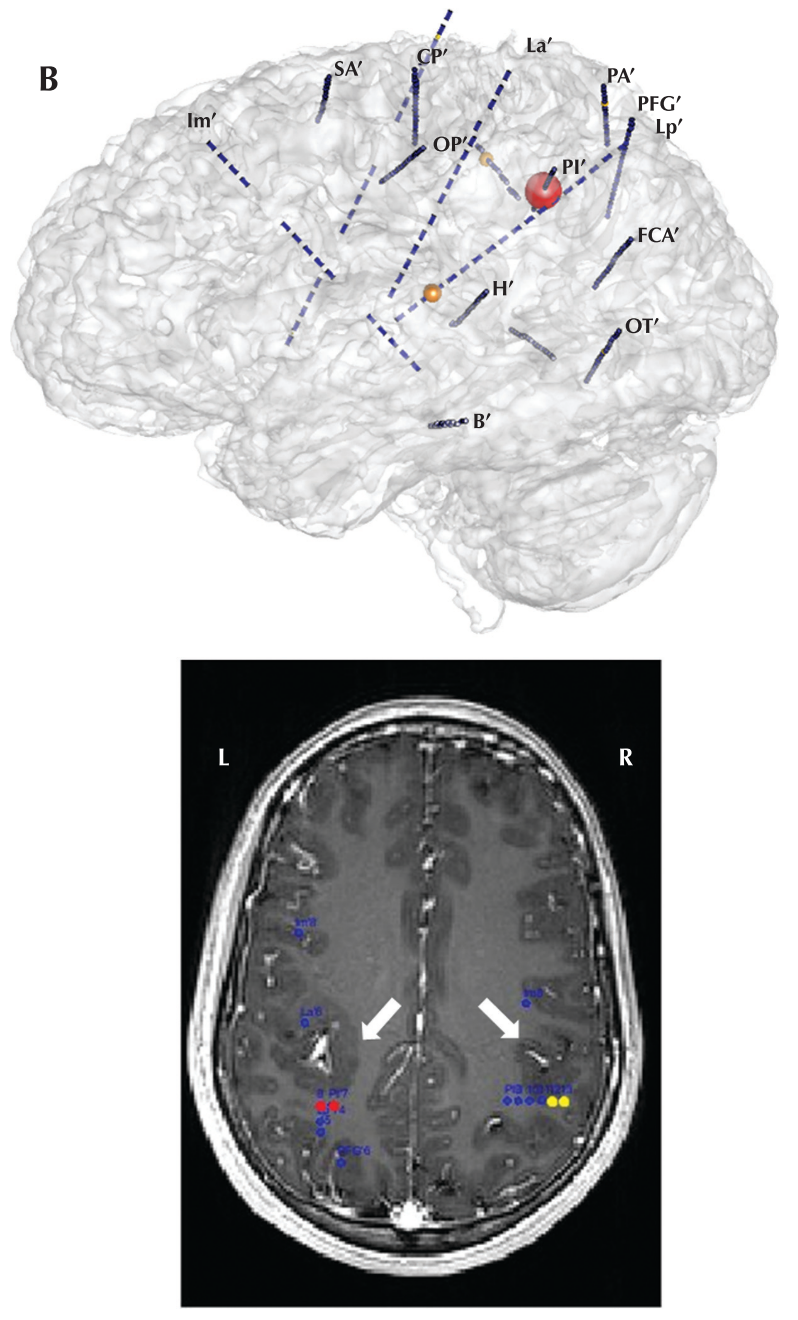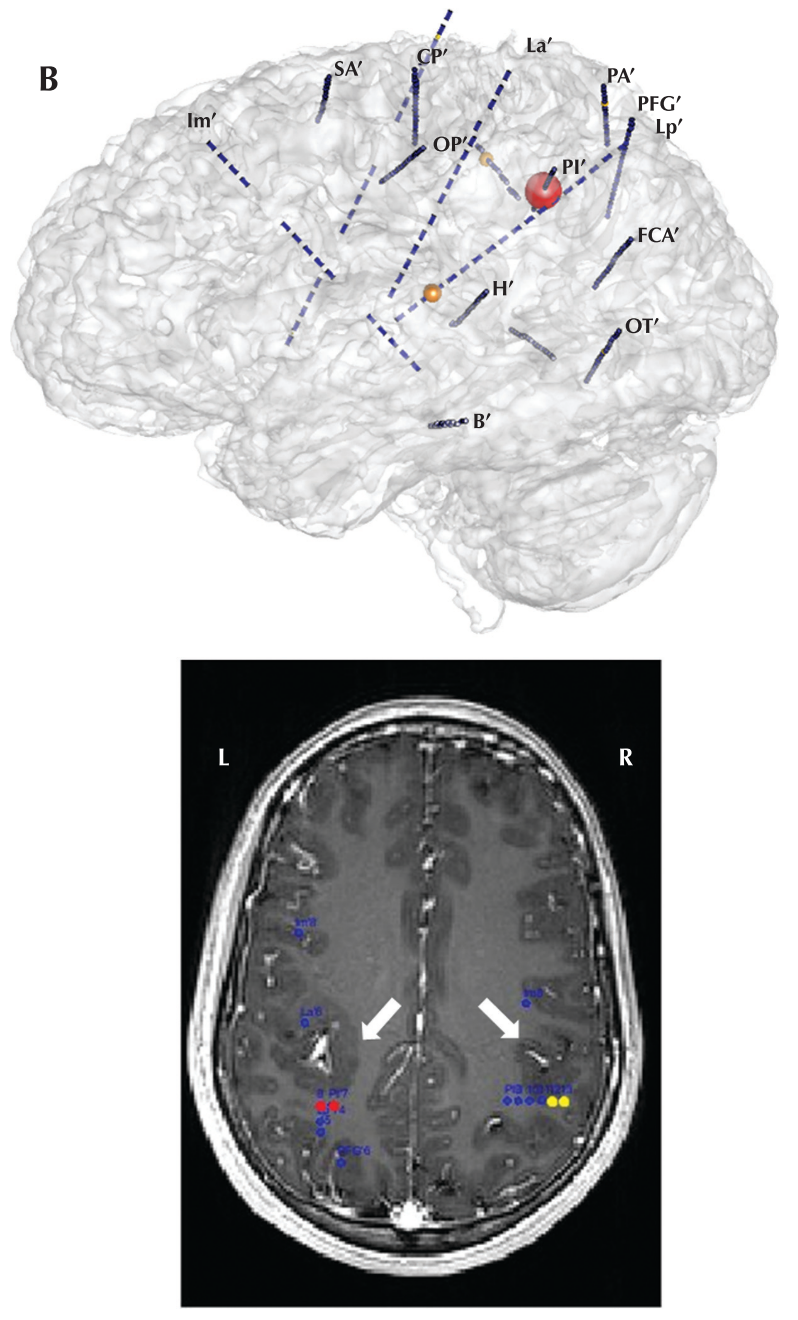 |
| 18 | 46 | male | right-handed | AH #6 | stimulation | Jacques Jonas et al，Neurol, 2014,#1 | O1-O2、O4-5 | 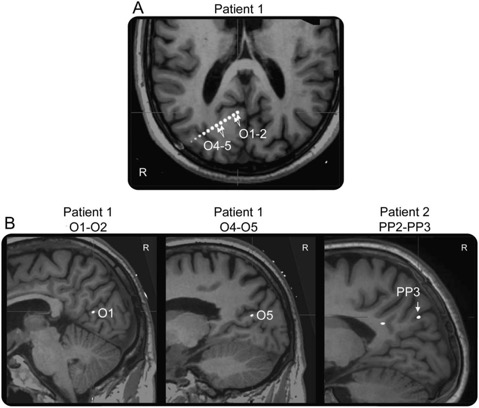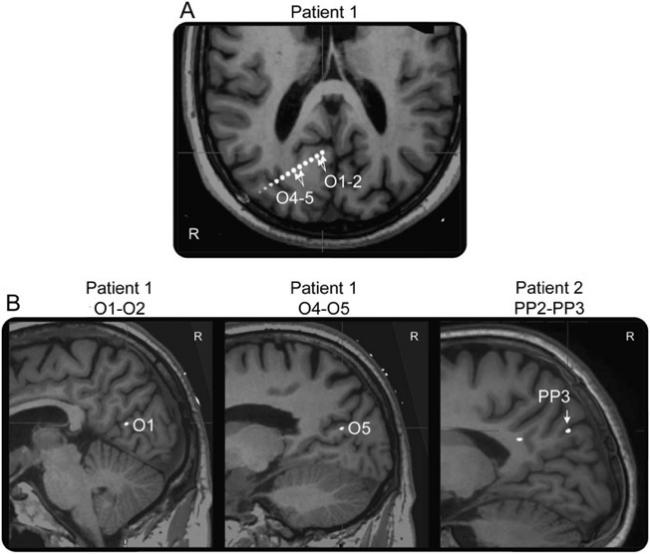 |
| 19 | 24 | female | right-handed | AH #7 | stimulation | Jacques Jonas et al，Neurol, 2014,#2 | PP2-PP3 | 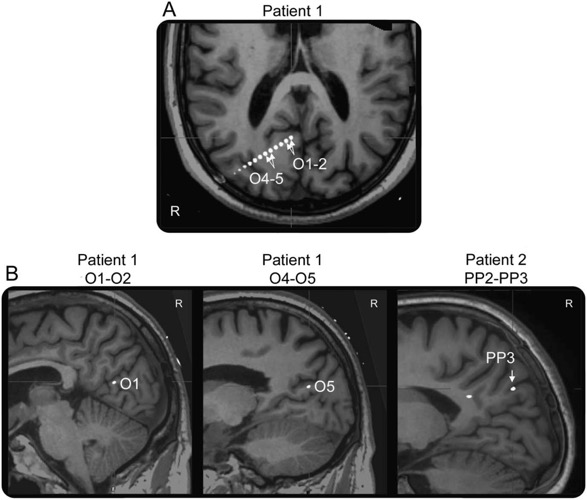 |
| 20 | 22 | female | right-handed | HAS#1 | lesion | Blanke O, et al, Brain, 2004,#2 cited by Heydrich L, et al, Brain. 2013,#4 Blanke O, et al, Brain, 2004,#2 cited by Heydrich L, et al, Brain. 2013,#4 | L_parieto-temporal cortex | 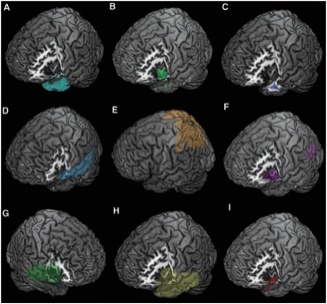 |
| 21 | 43 | female | right-handed | HAS#2 | lesion | Blanke O, et al, Brain, 2004,#5cited by Heydrich L, et al, Brain. 2013,#6 | two regions: L_angular gyrus & the lateral occipital gyrus; L_insula(L_fronto-temporal-insular cortex and the L_parieto-occipital cortex) | 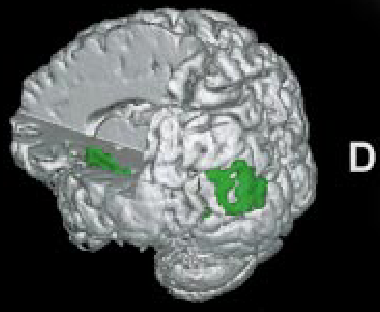 |
| 22 | 44 | male | right-handed | HAS#3 | lesion | Heydrich L, et al, Brain. 2013, #1 | L_temporal lobe | 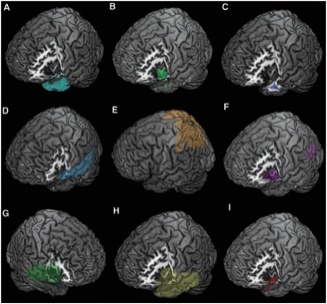 |
| 23 | 15 | female | right-handed | HAS#4 | lesion | Heydrich L, et al, Brain. 2013,#2 | L_medial temporal lobe | 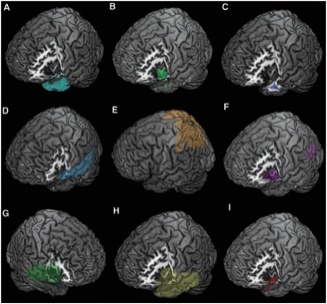 |
| 24 | 31 | male | right-handed | HAS#5 | lesion | Heydrich L, et al, Brain. 2013,#3 | L_temporal lobe | 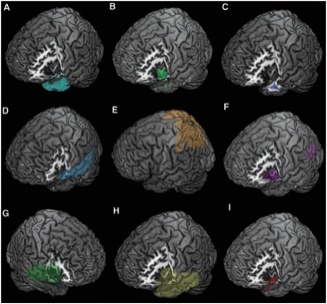 |
| 25 | 17 | girl | right-handed | HAS #6 | stimulation | Yu K, et al, J Clin Neurosci. 2018 | no available | 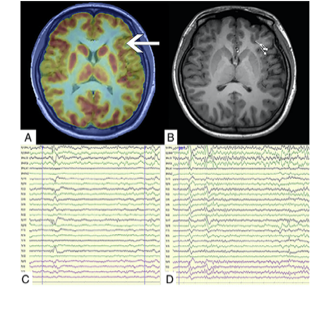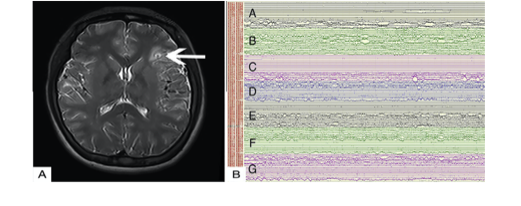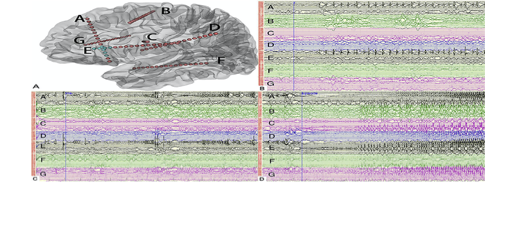 |

**Figure S1 Lesion network mapping of OBE, AH, and HAS**

**
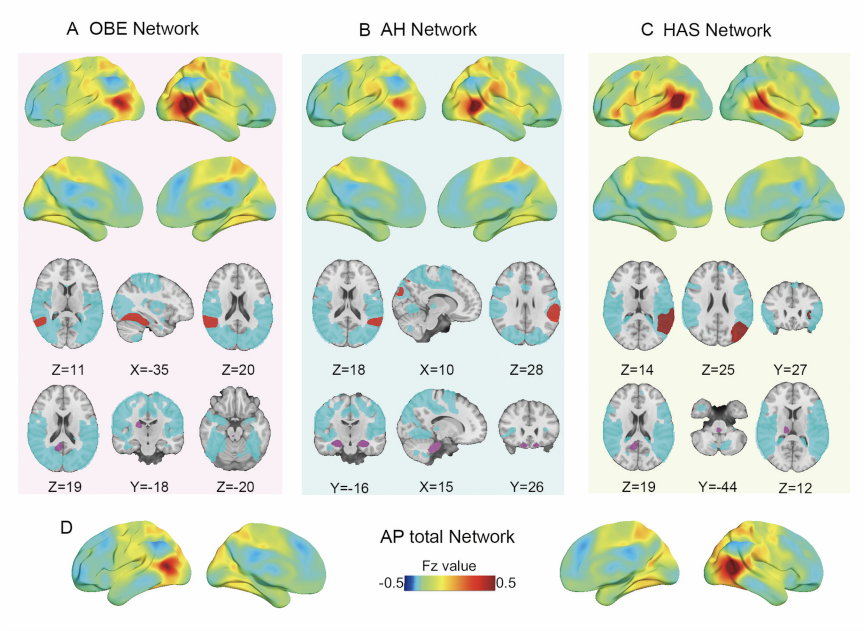
**

The network for each AP subtypes. (A) OBE network. (B) AH network. (C) HAS network. (D) AP total network.
